# Editorial: Trends in Neuroergonomics

**DOI:** 10.3389/fnhum.2017.00165

**Published:** 2017-04-05

**Authors:** Klaus Gramann, Stephen H. Fairclough, Thorsten O. Zander, Hasan Ayaz

**Affiliations:** ^1^Department of Psychology and Ergonomics, Berlin Institute of Technology (TU Berlin)Berlin, Germany; ^2^Center for Advanced Neurological Engineering, University of California, San DiegoSan Diego, CA, USA; ^3^School of Natural Sciences and Psychology, Liverpool John Moores UniversityLiverpool, UK; ^4^Team PhyPA, Berlin Institute of Technology (TU Berlin)Berlin, Germany; ^5^School of Biomedical Engineering, Science and Health Systems, Drexel UniversityPhiladelphia, PA, USA; ^6^Department of Family and Community Health, University of PennsylvaniaPhiladelphia, PA, USA; ^7^Division of General Pediatrics, Children's Hospital of PhiladelphiaPhiladelphia, PA, USA

**Keywords:** neuroergonomics, human-machine interaction, brain-computer interface, mobile brain/body imaging, physiological computing, EEG/ERP, NIRS/fNIRS, tDCS

## New methods in neuroergonomics

This Research Topic is dedicated to Professor Raja Parasuraman who unexpectedly passed on March 22nd 2015.

Raja Parasuraman's pioneering work led to the emergence of Neuroergonomics as a new scientific field. Neuroergonomics is defined as the study of the human brain in relation to performance at work and everyday settings (Parasuraman, 2003; Parasuraman and Rizzo, 2008). Since the advent of Neuroergonomics, significant progress has been made with respect to methodology and tools for the investigation of the brain and behavior at work. This is especially the case for neuroscientific methods where the availability of ambulatory hardware, wearable sensors, and advanced data analyses allow for imaging of brain dynamics in humans in applied environments.

For neuroergonomics, the application of brain imaging in real-world scenarios is highly desirable as an investigation tool. Traditionally, brain imaging experiments in human factors research tend to avoid active behavior for fear of artifacts contaminating the signal of interest. Here, the development of new data analyses techniques as well as the combination of different methods providing complementary insights into brain and behavioral dynamics allow for new insights into the human-machine interaction. To overcome the problem of artifactual data in mobile recordings and to allow analyses of brain activity in real working environments new portable sensors and improved analyses approaches have to be developed. Hence, deployment of portable neuroimaging technologies to real time settings could help assess cognitive and motivational states of personnel assigned to perform critical tasks.

## The “trends in neuroergonomics” research topic: a brief introduction

The eBook of this Frontiers Research Topic is divided into four sections, defined by the primary research methods used to address a variety of neuroergonomic research questions. The scientific topics range from air traffic control and automation, over mental load detection and the use of brain activity to control a system (brain computer interfaces, BCI), to physical work, rehabilitation, and training. Across the diverse research areas, the majority of studies in this Research Topic used electroencephalography (EEG), followed by functional near infrared spectroscopy (fNIRS), reflecting the constantly growing use of these methods in neuroergonomics. At the same time this eBook clearly demonstrates a trend to investigate physical and cognitive activity outside standard laboratory settings, moving neuroergonomics “into the wild.” In addition, traditional methods like the measurement of eye movements, pupil metrics, (ECG), and established imaging approaches like functional magnetic resonance imaging (fMRI) are used in combination with other methods to better understand the physiological responses to cognitive or physical tasks and their coupling to hemodynamic changes. The different sections include original research articles, but also reviews and opinion pieces. Here, we provide short summaries as an orientation for the interested reader.

The first section in this eBook thus comprises studies that used EEG to investigate ergonomic research questions with three studies using EEG in a mobile setting. The first of these by Jungnickel and Gramann uses a mobile brain/body imaging approach (MoBI; Makeig et al., 2009; Gramann et al., 2012, 2014) to investigate the brain and behavioral dynamics of human participants interacting with dynamically moving objects. The results indicate increased activity in parietal regions when active physical behavior as compared to standard laboratory button press behavior was required to respond to relevant changes in the environment. The findings point to changes in brain dynamic states dependent on the behavioral state. The study by Wascher et al. demonstrates that mobile EEG allows for a non-obtrusive assessment of mental fatigue in natural working situations. The authors investigate EEG variations time-locked to eye-blinks as a new tool to unobtrusively monitor cognitive processing in real-life environments. Mijović et al. also use EEG in a naturalistic work environment to show that instructed responses can increase attention as reflected in brain dynamics without changes in response parameters. The results point to the possibility to use instructed responses to increase attentional processing without compromising work performance in manual assembly tasks. Again, explicitly allowing movement of participants, Meinel et al. demonstrate that EEG can be used to improve motor rehabilitation approaches. Better performance in movement tasks can be achieved by identifying comodulation of different sources in the EEG before a movement is executed. The results provide participant-specific prediction of performance fluctuations that could be used to enhance neuroergonomic and rehabilitation scenarios. The last study by Zander et al. investigates how well a passive brain-computer Interface can work in an autonomous driving scenario using a dry EEG system. The results reveal comfort issues but acceptable usability of the tested EEG system and sufficient signal quality for use in an autonomous driving context.

A number of EEG-studies use the recorded brain electrical signals for system interaction through brain-computer interfaces (BCIs, Zander and Kothe, 2011). In this context, Kirchner et al. demonstrate that event-related potentials can be used on a single trial level to infer task-engagement of an operator controlling multiple robots and to adapt the man-machine interface to the individual operator. The results could be used to adapt the task load to operators with different qualifications or capabilities to avoid mental overload. Alonso-Valerdi et al. suggest that a wider variety of control commands in motor imagery-based BCIs might lead to an accelerate brain-computer communication while Callan et al. increase response speed in flight simulation by using a passive BCI based on MEG. The former study provides insights into the use of control commands to increase BCI-based system communication while the latter study demonstrates the potential to decode motor intention faster than manual control in response to hazardous change in the system interaction cycle. Roy et al. investigate mental workload based on auditory evoked potentials. The authors present a new minimal intrusive paradigm that paves the way to monitor operators' mental state in real-life settings to allow adaption of the user interface without interfering with the primary task. Caywood et al. increase the interpretability of BCI models by using the approach of Gaussian Process Regression for assessing cognitive workload. Ewing et al. describe the development of an adaptive game system that measures spontaneous EEG activity in real-time in order to adjust the difficulty level of the game. In two studies the concept of a biocybernetic control loop (Fairclough, 2009) is introduced in detail with a particular emphasis on validating EEG measures experimentally prior to their incorporation into an adaptive game system.

Studies using EEG in combination with other methods show that different physiological parameters can lead to an improved understanding of the construct under investigation. Ko et al. demonstrate the advantage of integrating the high resolution in the time and spatial domain for EEG and fMRI, respectively, in a stop-signal paradigm. Their results from multimodal recordings provide new insights into the complex brain networks underlying inhibitory control in naturalistic environments. Using EEG in combination with ECG and fNIRS, Ahn et al. investigate mental fatigue in drivers. They show that a combination of different physiological measures substantially improves the classification of sleep deprived or well-rested drivers. Scheer et al. investigate the demands on mental resources during a closed-loop steering task in simulated car driving scenarios. The results indicate an impact of steering demands on event-related EEG activity for task-irrelevant distractor probes allowing for an evaluation of mental workload in steering environments.

The second section summarizes the use of fNIRS in traditional stationary settings but also in new mobile applications. The first study of these, Von Lühmann et al. describe the development of a wireless and low-cost open source fNIRS hardware with details on system concept, hardware, software and mechanical implementation. The proposed system was tested in a mental arithmetic BCI experiment. Mandrick et al. discuss electrocortical and neurovascular measures with respect to the measurement of mental workload. They propose that EEG and fNIRS are complementary methods in the context of applied testing in the sense that the weakness of each approach, e.g., poor spatial and temporal resolution, respectively, is counteracted by the strength of the other. They argue for a combined fNIRS-EEG approach to index neurovascular coupling during the assessment of mental workload.

In Bediz et al. the effects of supramaximal exercise on cognitive task related oxygenation changes are investigated. Performance in a working memory task before and after exercise indicated higher task related activation changes in prefrontal cortex post-exercise and higher cognitive task related brain activation increase in high-performing participants. The study by Carrieri et al. investigates the neural correlates of a cognitive/motor task in a virtual reality (VR) environment. The findings support the use of VR in combination with fNIRS as a very good platform for neuroergonomic studies to objectively evaluate cortical hemodynamic activity. Mckendrick et al. utilize ultra-portable, wearable and miniaturized fNIRS sensors on participants walking outdoors in the open air. The battery-operated miniaturized system implementation was described by Ayaz et al. (2013). The results of a spatial navigation task indicated greater mental capacity reserves for users with head mounted displays but also unwanted attention capture and cognitive tunneling as indicated by hemodynamics measures. The final contribution in this section by Durantin et al. provides evidence for using Kalman filter as a suitable approach for real-time noise removal for fNIRS signals in ecological situations and the development of BCI. The findings from working memory tasks indicate Kalman filter increased the performance of the classification of task load levels based on brain signal.

Section three comprises studies using stimulation methods alone or in combination with other methods to investigate stimulation-based improvement of motor or cognitive functions. The introductory commentary by Besson et al. prepares the stage for this section by providing a critical commentary on existing transcranial direct current stimulation (tDCS) protocols to enhance neuroplasticity and enhance performance in real-world settings. They argue that priming tDCS protocols have significant potential to improve learning and motor performance. Callan et al. demonstrate the simultaneous use of tDCS and fMRI to investigate the effect of neurostimulation on resting state functional connectivity and behavioral performance. The results reveal greater spontaneous resting state activity for the tDCS group with higher resting state functional connectivity for participants demonstrating performance improvement in a visual search task. Choe et al. investigate the use of tDCS along with multimodal neuroimaging (EEG and fNIRS) demonstrating that tDCS stimulation to the dorsolateral prefrontal or left motor cortex during flight simulation training enhances behavioral performance and changes neurophysiological measurements (EEG and fNIRS) indicating improved skill acquisition consistent with previous studies (Ayaz et al., 2012). The final contribution in this section is the review of Teo et al. providing a discussion of the theoretical framework underlying the use of VR in combination with neuroimaging and neuromodulation as a therapeutic intervention for neurorehabilitation. The authors provide evidence for the use of VR in treating motor and mental disorders such as cerebral palsy, Parkinson's disease, stroke, schizophrenia, anxiety disorders, and other emerging clinical areas.

The fourth and last section in this eBook then covers research approaches using eye movement measures including pupilometric methods and other peripheral physiological methods like ECG. The first study in this section by Causse et al. uses EEG and pupilometry to investigate the impact of high working memory load on language processing during piloting. The results demonstrate high working memory load to disrupt visual and language processing and a subtle effect of congruency that was observable only at an electrophysiological level. In the study by Leff et al. the authors use a collaborative gaze channel (CGC) to detect and display trainer gaze behavior to trainees in surgery tasks. The results of a simulated robotic surgery task imply liberation of attentional resources with the use of CGC potentially improving the capability of trainees to attend to additional safety critical events during the procedure. Causse et al. then demonstrate that the pupil diameter correlates with inattentional deafness in an air traffic control task with varying perceptual and cognitive load.

The final contribution to this Research Topic is a review on neuroscientific methods in automation research by Drnec et al. The authors provide a comprehensive overview on neuroscientific methods in trust in automation research and summarize how neuroscience can improve interaction design.

## Concluding remarks

Neuroergonomics has demonstrated an incredible development since the introduction of the field by Raja Parasuraman. This Research Topic demonstrates how different methods can be used to better understand the mind, body, and brain at work and to create and design systems that are better adapted to and make use of the human information processing structures, including the body and the brain. This research is important, because it allows for a human-centered design of environments that include natural behaviors.

## Author contributions

All authors listed, have made substantial, direct and intellectual contribution to the work, and approved it for publication.

## Funding

This Research Topic gathers, among other contributions, papers presented at the 11th Berlin Workshop Human-Machine Systems, 2016 supported by a grant from the German Research Foundation (DFG GR2627/6-1) awarded to KG.

### Conflict of interest statement

The authors declare that the research was conducted in the absence of any commercial or financial relationships that could be construed as a potential conflict of interest.

## References

[B1] Ayaz H. Onaral B. Izzetoglu K. Shewokis P. A. McKendrick R. Parasuraman R. (2013). Continuous monitoring of brain dynamics with functional near infrared spectroscopy as a tool for neuroergonomic research: Empirical examples and a technological development. Front. Hum. Neurosci. 7, 1–13. 10.3389/fnhum.2013.00871 24385959 PMC3866520

[B2] Ayaz H. Shewokis P. A. Bunce S. Izzetoglu K. Willems B. Onaral B. (2012). Optical brain monitoring for operator training and mental workload assessment. Neuroimage 59, 36–47. 10.1016/j.neuroimage.2011.06.023 21722738

[B3] Fairclough S. H. (2009). Fundamentals of physiological computing. Interact. Comput. 21, 133–145. 10.1016/j.intcom.2008.10.011

[B4] Gramann K. Ferris D. P. Gwin J. Makeig S. (2014). Imaging natural cognition in action. Int. J. Psychophysiol. 91, 22–29. 10.1016/j.ijpsycho.2013.09.003 24076470 PMC3983402

[B5] Gramann K. Gwin J. T. Ferris D. P. Oie K. Jung T.-P. Lin C.-T.. (2012). Cognition in action: Imaging brain/body dynamics in mobile humans. Rev. Neurosci. 22, 593–608. 10.1515/RNS.2011.047 22070621

[B6] Makeig S. Gramann K. Jung T.-P. Sejnowski T. J. Poizner H. (2009). Linking brain, mind and behavior. Int. J. Psychophysiol. 73, 95–100. 10.1016/j.ijpsycho.2008.11.008 19414039 PMC2796545

[B7] Parasuraman R. (2003). Neuroergonomics: research and practice. Theor. Issues Ergon. Sci. 4, 5–20. 10.1080/14639220210199753

[B8] Parasuraman R. Rizzo M. (2008). Neuroergonomics: The Brain at Work. Oxford: Oxford University Press.

[B9] Zander T. O. Kothe C. (2011). Towards passive brain–computer interfaces: applying brain–computer interface technology to human–machine systems in general. J. Neural Eng. 8:025005. 10.1088/1741-2560/8/2/025005 21436512

